# Construction of an evaluation index system for nurse-led discharge planning competence in patients with chronic heart failure based on the iceberg model

**DOI:** 10.3389/fcvm.2026.1922348

**Published:** 2026-10-07

**Authors:** Jie Chen, Yuqing Wang, Haotian Zhang, Yuntao Yu, Haoxin Tian, Enyuan Yan, Guiyun Wang, Yanyan Fan

**Affiliations:** 1 School of Nursing, Shandong Medical and Pharmaceutical University, Yantai, China; 2 School of Nursing, Shandong Xiehe University, Jinan, China

**Keywords:** analytic hierarchy process, chronic heart failure, competency assessment, delphi method, discharge planning, nurse-led

## Abstract

**Objective:**

To construct an iceberg model-based competency evaluation index system for nurse-led discharge planning in patients with chronic heart failure (CHF), providing a scientific foundation for the cultivation and management of nurses’ discharge planning competencies.

**Methods:**

A preliminary item pool was established through a literature review. The Delphi method was employed to conduct three rounds of expert consultation with 17 experts. Following the three Delphi rounds, the same 17 experts were invited to complete pairwise comparison questionnaires based on the Saaty 1–9 scale, independently evaluating the relative importance of indicators at each hierarchical level. The research team aggregated all pairwise comparison matrices and computed indicator weights using the Analytic Hierarchy Process (AHP).

**Results:**

The effective response rates for all three rounds were 100%. The rates of experts proposing modifications were 64.70%, 29.41%, and 11.76%, respectively. The authority coefficient (Cr) was 0.93. Kendall's W coefficients for the first-, second-, and third-level indicators across the three rounds were 0.430, 0.308, and 0.158; 0.431, 0.321, and 0.292; and 0.365, 0.204, and 0.203, respectively. Moderate coordination was observed for first- and second-level indicators, while third-level indicators showed relatively weaker coordination; all differences were statistically significant (all *P* < 0.001). The final system comprised 5 first-level, 16 s-level, and 52 third-level indicators.

**Conclusion:**

The competency evaluation index system demonstrates strong scientific rigor, reliability, and practical utility. It provides a reference for the training, evaluation, and instructional design of nurse-led CHF discharge planning competencies, and offers an evidence-based foundation for healthcare institutions to develop relevant training programs and assessment criteria.

## Introduction

Chronic heart failure (CHF) is a clinical syndrome characterized by impaired cardiac pumping capacity resulting from structural and/or functional abnormalities, leading to a cascade of compensatory cardiac changes and inadequate organ perfusion (1). An estimated 64.3 million individuals worldwide are affected by CHF (2). Following discharge, patients frequently encounter recurrent symptoms (3), complex multimorbidity management (4), inadequate self-management capabilities (5), and poor treatment adherence, all of which contribute to elevated readmission rates (6). Discharge planning refers to a systematic process wherein multidisciplinary team members identify patients with high post-discharge care needs and formulate detailed discharge guidance to ensure a smooth transition from hospital to home or other care settings (7). As the healthcare professionals maintaining the most continuous patient contact, nurses play pivotal roles in assessment, education, communication and coordination, follow-up, and ongoing management (8). Lee et al. employed the Delphi method to establish core educational content for the hospital-to-home transition period in HF patients (9); Simpson et al. constructed a competency framework for heart failure specialist nurses in the United Kingdom in 2022, encompassing domains such as specialist knowledge, advanced clinical assessment, medication management, communication, and leadership, thereby providing an international reference for nurse-led HF transitional care competencies (10); Koontalay et al. identified five key elements of nurse-led CHF multidisciplinary services through a Delphi study (11). In China, researchers Lu et al. validated a core competency scale for nurse-led elderly discharge planning (12); Han et al. developed an empowerment-based transitional care protocol for CHF using the Delphi method (13).

However, existing research predominantly focuses on intervention outcomes and patient-centered endpoints, with related assessment tools primarily measuring patient discharge readiness. Insufficient attention has been devoted to delineating the competency components and evaluation criteria required for nurse-led discharge planning, and a context-specific competency evaluation instrument tailored to CHF discharge planning remains lacking. Therefore, constructing a competency evaluation index system for nurse-led CHF discharge planning would help clarify competency requirements and provide an evidence-based foundation for nurse training, assessment, and standardized management.

In summary, the existing literature predominantly addresses patient-side educational content or generic discharge planning competencies, leaving a notable gap: a nurse-led evaluation instrument grounded in the CHF context that encompasses both “visible” and “hidden” competencies through the lens of the iceberg model.

This study adopts the iceberg model as its theoretical framework—encompassing knowledge, skills, personal traits, self-concept, and intrinsic motivation—to address the aforementioned gap, thereby establishing the conceptual foundation of this research (14). Based on a systematic review of domestic and international literature, we extracted competency elements required for nurse-led CHF discharge planning, formed an initial indicator item pool, and conducted iterative revisions of the indicator content, nomenclature, and hierarchical attribution through three rounds of Delphi expert consultation. The resulting nurse-led CHF discharge planning competency evaluation index system was constructed through a rigorous methodological process with strong scientific validity (15).

## Research methods

### Establishment of the research team

The research team comprised 7 members, including 1 nursing education expert, 1 CHF clinical nursing specialist, and 5 nursing graduate students. Team responsibilities encompassed literature retrieval, indicator item pool construction, expert consultation questionnaire development, expert selection, questionnaire administration, and statistical data analysis.

### Theoretical framework

The iceberg model served as the theoretical foundation. Indicators were categorized into five dimensions—knowledge, skills, personal traits, self-concept, and intrinsic motivation—forming the initial framework for the nurse-led CHF discharge planning competency evaluation index system, providing a theoretical basis for subsequent item extraction, classification, and expert consultation.

### Ethical considerations

This study was approved by the Ethics Committee of Shandong Medical and Pharmaceutical University (Approval No.: 202505227). All participating experts electronically provided signed informed consent.

### Literature review

A systematic literature search was conducted across Web of Science, PubMed, The Cochrane Library, EMbase, CINAHL, CNKI, Wanfang, VIP, and CBM databases from inception to August 2025. Chinese search terms included: chronic heart failure, discharge planning, discharge guidance, discharge education, discharge readiness service, continuity of care, transitional care, nurse-led, nursing competency, and related terms. English search terms included: chronic heart failure, discharge planning, discharge instruction, continuity of care, transitional care, nurse-led, nursing competency, and related terms. Reference lists of included articles were manually screened for additional relevant indicators.

### Inclusion and exclusion criteria

Inclusion criteria: (1) the study population involved nurses or nursing teams leading discharge planning or CHF transitional care; (2) the study addressed competencies, skills, knowledge, or role behaviors; (3) peer-reviewed literature in English or Chinese. Exclusion criteria: studies solely measuring patient discharge readiness, lacking nurse competency items, or from which indicators could not be extracted. Two researchers independently extracted data, with discrepancies resolved by a third investigator. This process yielded an initial pool of 68 items.

### Expert selection

The Delphi method generally recommends recruiting 15–50 experts (13). For this study, 17 experts from 7 provinces were recruited, which meets the minimum sample size requirement while ensuring geographic diversity. The professional distribution of participants was as follows: 9 nursing administrators (52.9%), 4 clinical nurses, 2 nursing educators, and 2 physicians. The higher proportion of nursing administrators aligns with the reality that discharge planning in China is typically coordinated by nursing departments at the institutional level. Administrators can provide a systems-level perspective on the required competencies, while clinical nurses offer insights into implementation details; this tripartite composition helps enhance the content validity of the study (16).

### Three-round procedure and scoring summary

Round 1: The 68-item pool plus an open comment box was distributed. Summary metrics for each item included mean ($\bar{x}$), standard deviation (SD), coefficient of variation (CV), and full-score rate (proportion of 5-point ratings); open-ended suggestions were transcribed verbatim. Retention criteria: mean >3.5 and CV < 0.25 (17); 11 experts (64.7%) provided comments. Round 2: The revised 56 items from Round 1 were redistributed, reporting the same metrics (mean/CV/full-score rate) along with feedback noting “how your Round 1 suggestions were addressed.” Five experts (29.4%) provided further comments. Round 3: 52 items were distributed, applying the same retention criteria (mean > 3.5, CV < 0.25) for confirmation without introducing new items. Two experts (11.8%) suggested minor wording adjustments. All open comments were managed through a coding sheet organized by “comment—assigned indicator—suggestion type (modify/delete/merge/add)—team decision,” with two independent coders and weekly team review; rejected suggestions were documented with rationale (e.g., “overlaps with I-1”).

### Expert consultation

#### Determine the selection criteria of experts

Inclusion criteria:

① Nursing education experts: Those who have been engaged in nursing teaching or scientific research related to heart failure for more than 5 years, hold a master’s degree or above, have the title of associate professor or above, and are familiar with the content related to the discharge plan for heart failure patients.
② Clinical heart failure nursing experts: Those who have been working for more than 5 years, hold a bachelor’s degree or above, have the title of nurse-in-charge or above, and are familiar with the content of discharge planning work.
③ Nursing management experts: Those who have been engaged in nursing management in a tertiary hospital for more than 5 years, hold a bachelor’s degree or above, have the title of nurse-in-charge or above, and are familiar with the discharge planning for patients with heart failure.
④ Clinical medical experts: Those who have been working for more than 5 years, are engaged in medical work related to heart failure, hold a master’s degree or above, have the title of associate chief physician or above, and are familiar with the content of discharge planning work.
⑤ Informed consent to participate in this study.

Exclusion criteria: ① Unable to respond to the questionnaire on time; ② There were large - area missed answers or obvious logical contradictions in the same round of questionnaires.

#### Form the expert letter consultation questionnaire

On the basis of literature research, this study initially established the evaluation index system for the nurse - led discharge planning ability for patients with chronic heart failure and compiled an expert consultation questionnaire. The questionnaire consists of 4 parts.

- Letter to experts: It includes the background, purpose, and significance of the study, as well as the method of filling out the questionnaire.
- General information of experts: It includes gender, age, education, professional title, position, work unit, main work category, and years of working in related fields.
- Evaluation index system for the nurse - led discharge planning ability for patients with chronic heart failure indicators: Experts were invited to score the importance of each indicator. 5 points represent “very important”, 4 points represent “relatively important”, 3 points represent “generally important”, 2 points represent “not very important”, and 1 point represents “very unimportant”. Open - ended questions were set up to solicit suggestions for revision.
- Expert judgment basis and familiarity: It includes expert familiarity and judgment basis. Familiarity is divided into 5 levels. The judgment basis includes four aspects: theoretical analysis, practical experience, reference, and intuitive judgment, and each aspect is divided into three levels: large, medium, and small.

### Three-round delphi procedure

Electronic questionnaires were administered across three rounds. After each round, experts’ ratings and comments were compiled, analyzed, and discussed by the research team to generate the subsequent round's questionnaire. Additionally, following the third round, pairwise comparison questionnaires based on the Saaty 1–9 scale were distributed to the same 17 experts, who independently evaluated the relative importance of indicators at each hierarchical level. A total of 17 valid pairwise comparison questionnaires were returned, yielding a 100% response rate. The indicator retention criteria were mean importance score > 3.5 and coefficient of variation (CV) < 0.25 (18).

### Statistical methods

Data were processed using Excel and SPSS 26.0. Means, standard deviations, and CVs described the central tendency of expert ratings. Effective response rates reflected expert enthusiasm. The authority coefficient (Cr) was calculated as the arithmetic mean of the judgment basis coefficient (Ca) and the familiarity coefficient (Cs). Kendall's W coefficient ranges from 0 to 1, with values closer to 1 indicating higher expert agreement. Reference standards: W < 0.3 indicates weak agreement, 0.3–0.5 indicates moderate agreement, and > 0.5 indicates strong agreement (15); For the AHP weight calculation: the research team aggregated pairwise comparison results from 17 experts to construct group decision matrices at each hierarchical level, employed the geometric mean method to integrate individual judgments, computed the maximum eigenvalues and corresponding eigenvectors, and derived normalized weights. Consistency ratios (CR) < 0.10 were considered acceptable. Statistical significance was set at *P* < 0.05.

## Results

### General information of the letter expert

In this study, a total of 17 experts from Shandong, Gansu, Liaoning, Shanghai, Beijing, Yunnan, and Hunan were invited for correspondence consultation.

The age distribution of the experts was as follows: 4 (23.5%) were aged 31–40 years old, 11 (64.7%) were aged 41–50 years old, and 2 (11.8%) were over 50 years old.

Regarding their educational backgrounds: 8 (47.1%) had a bachelor’s degree, 6 (35.3%) had a master’s degree, and 3 (17.6%) had a doctor’s degree.

In terms of professional titles: 4 (23.5%) held intermediate titles, 8 (47.1%) held associate - senior titles, and 5 (29.4%) held senior titles.

Concerning working years: 3 (17.6%) had 11–15 years of work experience, 5 (29.4%) had 16–20 years of work experience, 3 (17.6%) had 21–25 years of work experience, and 6 (35.3%) had more than 25 years of work experience.

Regarding the professional structure: 4 (23.5%) were in clinical nursing, 2 (11.8%) were in nursing education, 9 (52.9%) were in nursing management, and 2 (11.8%) were in clinical medicine. See Table 1 for details.

**Table 1:** General information of experts (*n* = 17).

Projects	Categories	Number of people	Percentage
Age	31–40 years old	4	23.5%
	41–50	11	64.7%
	>50 years old	2	11.8%
Education	Undergraduate	8	47.1%
	Master's	6	35.3%
	PhD	3	17.6%
Title	Intermediate	4	23.5%
	Deputy Senior	8	47.1%
	Full Senior	5	29.4%
Years of service	11–15 years	3	17.6%
	16–20 years	5	29.4%
	21–25 years	3	17.6%
	>25 years	6	35.3%
Professional structure	Clinical nursing	4	23.5%
	Nursing education	2	11.8%
	Nursing administration	9	52.9%
	Clinical medicine	2	11.8%

### Expert positivity

A total of 17 questionnaires were distributed and 17 were recovered in the three - round consultation, and the effective recovery rate of the questionnaires was 100.00%. In the first round, 11 experts (64.70%) put forward suggestions for revision; in the second round, 5 experts (29.41%) proposed revisions; in the third round, 2 experts (11.76%) proposed revisions, indicating that the experts in this study were highly active.

### Degree of expert authority

In the three - round expert consultation of this study, the judgment basis coefficient of 17 experts was 0.91, the familiarity coefficient was 0.94, and the authority coefficient was 0.93, which showed that the authority of the experts was high (16).

### Concentration of experts’ opinions

In the first round of expert consultation, the minimum mean of the importance score for each index was 4.29, the minimum full - score rate was 47.06%, and the maximum standard deviation was 0.77. In the second round of expert consultation, the minimum mean value of the importance score for each index was 4.41, the minimum full - mark rate was 41.18%, and the maximum standard deviation was 0.51. In the third round of expert consultation, the minimum mean value of the importance score for each index was 4.59, the minimum full - score rate was 58.82%, and the maximum standard deviation was 0.51. With the increase in consultation rounds, the experts’ evaluations of each index gradually became more concentrated, and the consistency was good. So, the expert consultation was concluded (19).

### Coordination degree of expert opinions

In the first round of expert consultation, the coefficient of variation of each index was 0.00–0.23, and the Kendall’s W of the first - level, second - level, and third - level indicators was 0.430, 0.308, and 0.158, respectively.

In the second round of expert letter consultation, the coefficient of variation of each index was 0.00–0.12, and the Kendall’s W of the first - level, second - level, and third - level indicators was 0.431, 0.321, and 0.292, respectively.

In the third round of expert consultation, the coefficient of variation of each index ranged from 0.00 to 0.11, and the Kendall's W of the first - level, second - level, and third - level indicators was 0.365, 0.204, and 0.203, respectively. The differences were statistically significant (all *P* < 0.001). First- and second-level indicators demonstrated moderate coordination, while third-level indicators showed weaker coordination, potentially attributable to the larger number of third-level items and greater inter-expert variability in assessing specific sub-items. Subsequent large-sample empirical research will further optimize item coordination (20) (Table 2).

**Table 2:** *Kendall's W* coefficient and significance test results of three rounds of expert opinions.

Round of rounds	Indicators	*Kendall's* W	Chi-square value	*P-value*
First round	First-level indicators	0.430	29.268	<0.001
	Secondary indicators	0.308	62.742	<0.001
	Tertiary indicators	0.158	123.636	<0.001
Second round	First-level indicators	0.431	29.280	<0.001
	Secondary indicators	0.321	81.822	<0.001
	Tertiary indicators	0.292	262.816	<0.001
Third round	First-level indicators	0.365	24.800	<0.001
	Secondary indicators	0.204	51.940	<0.001
	Third-level indicators	0.203	176.066	<0.001

### Results of index revision

#### Modified indicators

(1) In the first round of expert consultation, based on the experts’ suggestions regarding the modification of evaluation indicators, the following suggestions were adopted after discussion by the research group:
  (1) The connotation of the level - 1 index “IV self - concept” was modified and adjusted to “nurses’ identity of their own professional values and professional roles in the process of leading the implementation of discharge plans for chronic heart failure”, in order to enhance its differentiation from “III personal characteristics” and “V intrinsic motivation”.
  (2) The secondary indicators of the knowledge dimension were reconstructed. The original “I - 1 chronic heart failure specialist knowledge” and “I - 2 discharge planning knowledge” were split into “I - 1 chronic heart failure clinical diagnosis and treatment knowledge, I - 2 discharge planning related risk prevention and continuous care knowledge, and I - 4 chronic heart failure nursing practice knowledge”, so as to reduce item repetition and enhance the systematicity and pertinence of the knowledge structure.
  (3) The secondary indicators of the skill dimension were modified. The original skill item “II - 1 assessment ability” was revised to “II - 1 advanced clinical assessment and judgment skills”, the original “II - 3 communication and coordination ability” was revised to “II - 2 cross - system coordination and communication skills”, and the original “II - 2 health education ability” was revised to “II - 3 patient and family education and empowerment skills”. Content such as caregiver training, follow - up arrangements, and quality tracking was included in the corresponding indicators.
  (4) The personal trait dimension was adjusted. The original “III - 1 respect, III - 2 responsibility, III - 3 empathy, III - 4 stress resistance” was revised to “III - 1 cognitive and thinking traits, III - 2 interpersonal and emotional traits”, so that the connotation of the dimension was more focused on thinking characteristics and emotional characteristics.
  (5) The self - concept dimension was restructured, and the second - level indicators were modified to “IV - 1 professional values, IV - 2 identity and role identity”, highlighting the value orientation and role orientation of nurses in leading the discharge plan of patients with chronic heart failure.
  (6) The intrinsic motivation dimension was modified. The original “V - 1 leadership, V - 2 active learning ability” was modified to “V - 1 achievement orientation and professional pursuit, V - 2 sense of responsibility and leadership, V - 3 ethical consciousness and justice”, so that the intrinsic motivation dimension index could better reflect the internal motivation of nurses’ continuous investment and active practice.
  (7) Some three - level indicators were modified and integrated. For example, “II - 1 - 1 capacity status assessment” was extended to “II - 1 - 1 comprehensive chronic heart failure physiological state assessment”, “II - 1 - 2 disease risk assessment” was revised to “II - 1 - 3 discharge risk assessment and grading”, and “V - 1 capacity status assessment” was revised to “II - 1 - 3 discharge risk assessment and grading”. Items related to communication, follow - up, caregiver education, device therapy, evidence - based practice, etc., were expressed and optimized.
(2) In the second round of expert consultation, the following modification suggestions were adopted after the research group's discussion:
  (1) “I - 4 Knowledge related to chronic heart failure nursing practice” was revised to “I - 4 Knowledge of advanced nursing practice and humanistic literacy”;
  (2) “I - 4 - 4 Knowledge of chronic disease psychology” was revised to “I - 4 - 3 Knowledge of nursing psychology”.
(3) In the third round of expert consultation, “II - 3 Education and empowerment skills for patients and family caregivers” was revised to “II - 3 Education and empowerment skills for patients and caregivers”, and the wording of “ability” and “initiative” in some items was unified.

#### Delete indicators

According to the calculation results of evaluation indicators, there is no need to delete indicators based on statistical screening criteria. However, based on expert opinions and research group discussions, some items with unclear meanings or improper assignments were deleted.

1. Deletion details in each round of expert consultation

First round: After the research group's discussion in the first round of expert consultation, some deletion suggestions proposed by experts were adopted. Specifically, the items with unclear expressions and directions in “II - 3 - 1 emotional dialogue” were deleted.

Second round: Delete the “I - 1 - 4 chronic heart failure complications and comorbidity management knowledge”. According to experts, this item does not belong to the core category of “clinical diagnosis and treatment knowledge of chronic heart failure”.

Third round: No deletion suggestions were added in the third round of expert consultation.

#### Combined indicators

1. Combination in the first round of expert consultation

In the first round of expert consultation, the original “IV - 2 reflective ability” and “V - 1 leadership” were merged into “II - 5 quality Improvement and Professional leadership”.

(1) Combination in the second round of expert consultation

“Pathogenesis and development rule of I - 1 - 1 chronic heart failure” and “knowledge of clinical manifestations and signs of I - 1 - 2 chronic heart failure” were merged into “etiology and clinical manifestations of I - 1 - 1 chronic heart failure”.

“IV - 1 - 4 ethics” and “IV - 1 - 5 adherence to fairness and justice” were merged. Experts believed that “fairness and justice” is an important part of ethical principles in nature, and it was suggested to be merged into the ethical items.

#### Add indicators

(1) In the first round of expert consultation, the following contents were revised after the discussion of the research group:
  (1) The index “I - 3 Health education and behavioral science knowledge” was added to emphasize that nurses should have the knowledge base for implementing health education and promoting patient behavior change when carrying out discharge planning for CHF patients.
  (2) The contents related to discharge plan making and management were added, and “II - 4 Plan making and management skills” was set in the skill dimension.
  (3) Content related to caregiver support was added, and “II - 3 Patient and family education and empowerment skills” was set in the skill dimension.
  (4) Content related to professional values was added, and “IV - 1 Professional values” was set under the dimension of self - concept.
  (5) The knowledge of chronic heart failure - related diseases was increased and refined into “I - 1 - 1 Pathogenesis and development law of chronic heart failure”, “I - 1 - 2 Knowledge of clinical manifestations and signs of chronic heart failure”, “I - 1 - 4 Knowledge of complications and comorbidity management”.
  (6) The knowledge of device therapy was increased, and the supplement of the left heart assist device was included in “I - 1 - 5 Knowledge related to chronic heart failure device therapy”.
(2) In the second round of expert consultation, “IV - 1 - 5 Abide by the guidelines of Caution and Independence” was added to emphasize the professional self - discipline requirements for nurses’ ability to adhere to standardized operations, ensure medication safety, and keep accurate records under unsupervised circumstances. In the third round of expert consultation, no new indicators were added.

The addition, deletion, merging, and modification of the above indicators were a dynamic adjustment process in each round of expert consultation. Some index names and numbers changed during the revision process. The final index name, hierarchical structure, and number are subject to the results shown in Table 3.

**Table 3:** Results of expert consultation on evaluation index system of nurse-led discharge planning ability for patients with chronic heart failure.

Indicators	Importance (x ± s, score)	Coefficient of Variation	Weights	Composite Weight
I Knowledge	5.00 ± 0.00	0.00	0.295	0.295
I-1 Clinical diagnosis and treatment knowledge of chronic heart failure	5.00 ± 0.00	0.00	0.286	0.084
I-1-1 Etiology and clinical manifestations of chronic heart failure	5.00 ± 0.00	0.00	0.539	0.045
I-1-2 Knowledge of chronic heart failure pharmacotherapy (specifically regarding dosing, indications, adverse effects, medication guidance, and adherence assessment for diuretics; ACEIs/ARBs/ARNIs; beta-blockers; MRAs; and SGLT2is)	5.00 ± 0.00	0.00	0.297	0.025
I-1-3 Knowledge related to device therapy for chronic heart failure	5.00 ± 0.00	0.00	0.164	0.014
I-2 discharge plan related risk prevention and continuous care knowledge	5.00 ± 0.00	0.00	0.286	0.084
I-2-1 Discharge planning theory and process knowledge	5.00 ± 0.00	0.00	0.443	0.037
I-2-2 Knowledge of discharge outcomes, readmission risk assessment and prediction tools	4.88 ± 0.33	0.07	0.387	0.033
I-2-3 Knowledge of out-of-hospital continuous care and social support resources	4.53 ± 0.51	0.11	0.170	0.014
I-3 Knowledge of Health education and behavioral Science	4.76 ± 0.44	0.09	0.143	0.042
I-3-1 Knowledge of health education	4.88 ± 0.33	0.07	0.329	0.014
I-3-2 Knowledge of Home Care and Cultural Health Literacy	4.53 ± 0.51	0.11	0.142	0.006
I-3-3 Theoretical knowledge of self-management support and behavior change	4.88 ± 0.33	0.07	0.329	0.014
I-3-4 Knowledge of motivational interviewing and communication skills	4.53 ± 0.51	0.11	0.142	0.006
I-4 Knowledge of advanced nursing practice and humanistic literacy	4.88 ± 0.33	0.07	0.286	0.084
I-4-1 Knowledge of nursing ethics and law	4.76 ± 0.44	0.09	0.141	0.012
I-4-2 Knowledge of nursing quality Improvement and evidence-based practice	5.00 ± 0.00	0.00	0.233	0.020
I-4-3 Knowledge of leadership and teamwork	5.00 ± 0.00	0.00	0.394	0.033
I-4-4 Knowledge of nursing psychology	5.00 ± 0.00	0.00	0.233	0.020
II Skills	5.00 ± 0.00	0.00	0.295	0.295
II-1 Advanced Clinical Assessment and Judgment Skills	4.82 ± 0.40	0.08	0.252	0.074
II-1-1 Comprehensive assessment of the physiological state of chronic heart failure	4.88 ± 0.33	0.07	0.141	0.010
II-1-2 Comprehensive assessment of mental state in chronic heart failure	4.94 ± 0.24	0.05	0.233	0.017
II-1-3 Discharge risk assessment and grading	5.00 ± 0.00	0.00	0.394	0.029
II-1-4 Assessment of medication safety	4.94 ± 0.24	0.05	0.233	0.017
II-2 Coordination and communication skills across systems	4.88 ± 0.33	0.07	0.106	0.031
II-2-1 Interdisciplinary team leadership and coordination	4.94 ± 0.24	0.05	0.312	0.010
II-2-2 System Navigation and resource links	4.59 ± 0.51	0.11	0.198	0.006
II-2-3 Structured handoff Communication	5.00 ± 0.00	0.00	0.490	0.015
II-3 Patient and Caregiver Education and empowerment Skills	4.76 ± 0.44	0.10	0.076	0.022
II-3-1 Individualized and adaptable educational skills	4.88 ± 0.33	0.07	0.297	0.007
II-3-2 Communication skills to promote patient behavior change	4.53 ± 0.51	0.11	0.164	0.004
II-3-3 Primary Caregiver training and Support	5.00 ± 0.00	0.00	0.539	0.012
II-4 Plan Development and management skills	5.00 ± 0.00	0.00	0.378	0.112
II-4-1 Development of a structured, individualized discharge plan	5.00 ± 0.00	0.00	0.400	0.045
II-4-2 Predictive risk identification and Alternative plan Development	5.00 ± 0.00	0.00	0.400	0.045
II-4-3 Task decomposition and implementation of discharge planning	4.88 ± 0.33	0.07	0.200	0.022
II-5 Quality Improvement and Professional Leadership	4.76 ± 0.44	0.10	0.189	0.056
II-5-1 Follow-up Tracking and Outcome Management	4.88 ± 0.33	0.07	0.297	0.017
II-5-2 Evidence-based Practice and process optimization	4.59 ± 0.51	0.11	0.164	0.009
II-5-3 Professional Guidance and Counseling	4.88 ± 0.33	0.07	0.539	0.030
III Personal Traits	4.59 ± 0.51	0.11	0.088	0.088
III-1 Cognitive and Thinking Traits	4.65 ± 0.50	0.11	0.333	0.029
III-1-1 Systematic thinking	4.88 ± 0.33	0.07	0.500	0.015
III-1-2 Forward thinking	4.88 ± 0.33	0.07	0.250	0.007
III-1-3 Pragmatic problem-solving orientation	4.88 ± 0.33	0.07	0.250	0.007
III-2 Interpersonal and Emotional Traits	4.88 ± 0.33	0.07	0.667	0.059
III-2-1 Calm and emotional stability	4.94 ± 0.24	0.05	0.312	0.018
III-2-2 Resolute advocacy	4.65 ± 0.49	0.11	0.198	0.012
III-2-3 Deep Empathy and Emotional Boundaries	4.82 ± 0.39	0.08	0.490	0.029
IV Self-concept	4.82 ± 0.39	0.08	0.140	0.140
IV-1 Professional values	5.00 ± 0.00	0.00	0.500	0.070
IV-1-1 is patient-centered	5.00 ± 0.00	0.00	0.193	0.014
IV-1-2 Pursuit of professional excellence and evidence-based practice	5.00 ± 0.00	0.00	0.193	0.014
IV-1-3 focuses on collaboration and leadership	4.88 ± 0.33	0.07	0.142	0.010
IV-1-4 Follow ethical principles	5.00 ± 0.00	0.00	0.277	0.019
IV-1-5 Adhere to the code of caution	4.76 ± 0.44	0.09	0.098	0.007
IV-1-6 Respect	4.76 ± 0.44	0.09	0.098	0.007
IV-2 Identity and Role identity	5.00 ± 0.00	0.00	0.500	0.070
IV-2-1 Professional Identity	5.00 ± 0.00	0.00	0.400	0.028
IV-2-2 Dominant role identity	5.00 ± 0.00	0.00	0.400	0.028
IV-2-3 Advanced Practice Role Identity	4.88 ± 0.33	0.07	0.200	0.014
V Intrinsic motivation	4.88 ± 0.33	0.07	0.182	0.182
V-1 Achievement orientation and professional pursuit	4.94 ± 0.24	0.05	0.297	0.054
V-1-1 Professional aggressiveness	5.00 ± 0.00	0.00	0.500	0.027
V-1-2 Proactive improvement and innovation	4.88 ± 0.33	0.07	0.500	0.027
V-2 Sense of responsibility and leadership	5.00 ± 0.00	0.00	0.539	0.098
V-2-1 Dominant role motivation	5.00 ± 0.00	0.00	0.490	0.048
V-2-2 Decision making and accountability for results	5.00 ± 0.00	0.00	0.312	0.031
V-2-3 Motivation to fight for resources for patients	4.82 ± 0.39	0.08	0.198	0.019
V-3 Ethical consciousness and sense of justice	4.76 ± 0.44	0.10	0.164	0.030
V-3-1 Ethical Adherence to Motivation	5.00 ± 0.00	0.00	0.667	0.020
V-3-2 Honesty and error correction	5.00 ± 0.00	0.00	0.333	0.010

#### Final indicator system and weights

After three rounds of expert letter consultation, the final evaluation index system for nurse - led discharge planning ability of patients with chronic heart failure was formed. It includes five first - level indicators, 16 s - level indicators, and 52 third - level indicators.

The analytic hierarchy process was used to calculate the weights of indicators at all levels, and a consistency test was carried out. The CR values of judgment matrices at all levels were all less than 0.10. The weights of first - level indicators were as follows: knowledge 0.295, skills 0.295, intrinsic motivation 0.182, self - concept 0.140, personal traits 0.088. The combination weights of the second - level indicators ranged from 0.022 to 0.112, and the combination weights of the third - level indicators ranged from 0.004 to 0.048. See Table 3 for details.

This system supports multiple: (1) as a specialist nurse training framework, the full 52-item set can inform curriculum design and competency benchmarking; (2) for position accreditation or performance evaluation, a subset of 18 high-weight indicators (combined weight > 0.02) may be selected for streamlined use; (3) as a self-assessment tool, a 5-point Likert scale (1 = not at all competent, 5 = fully competent) can yield both total scores and dimension-specific scores to identify competency gaps. Subsequent research will establish scoring cutoffs and interpretation standards for different use scenarios.

## Discussion

### Scientific rigor and reliability

Among the 17 consulting experts, 76.5% held associate senior or higher titles, and 52.9% possessed master's or doctoral degrees, with representation spanning clinical nursing, nursing education, nursing administration, and clinical medicine. The 100% response rate across all three rounds indicated strong expert engagement. The authority coefficient of 0.93 suggested high credibility of expert judgments. Kendall's W values for Rounds 1–3 were 0.430/0.308/0.158, 0.431/0.321/0.292, and 0.365/0.204/0.203, respectively (all *P* < 0.001), with progressively fewer experts proposing modifications, indicating converging consensus and stabilization of the indicator system (21, 22).

### Significance of the iceberg model framework

CHF is characterized by prolonged disease course, marked symptom fluctuations, multimorbidity, and high readmission risk. Effective discharge planning is essential for bridging inpatient treatment and outpatient care (23–25). Previous studies have established that nurses in CHF discharge planning assume responsibilities extending beyond health education and follow-up guidance to encompass risk assessment, multidisciplinary coordination, resource linkage, and continuity of care (26–28). The final system comprises 5 first-level, 16 s-level, and 52 third-level indicators. Knowledge and Skills received the highest equal weights (0.295 each), underscoring expert consensus that both constitute foundational competencies for nurse-led CHF discharge planning—consistent with Simpson et al.'s emphasis on specialist knowledge and practical competencies within their heart failure nurse competency framework (10). CHF discharge planning spans inpatient assessment, plan formulation, health education, and post-discharge follow-up, demanding not only robust specialty knowledge but also the capacity to translate knowledge into practice for risk identification, care planning, communication, and continuity management (29–31). Intrinsic motivation ranked third (0.182), highlighting that responsibility, leadership initiative, achievement orientation, and ethical awareness are critical drivers of sustained, high-quality discharge planning. Self-concept ranked fourth (0.140), suggesting that professional values and role identity influence nurses’ initiative and accountability (32). Although personal traits received the lowest weight (0.088), they remain fundamental to risk judgment, communication, and emotional regulation—indicating that experts valued not only whether nurses can perform tasks but also how well they perform them.

Among second-level indicators, higher weights were assigned to CHF clinical diagnosis and treatment knowledge, planning and management skills, and responsibility and leadership initiative—reflecting expert consensus that nurses must possess both problem-identification capabilities and the capacity to formulate individualized plans and actively facilitate care transitions across hospital-community boundaries (33, 34). This comprehensive system provides a reference for competency cultivation, position evaluation, and quality improvement initiatives.

### Clinical utility

This system encompasses five dimensions—knowledge, skills, personal traits, self-concept, and intrinsic motivation—capturing both visible and hidden competencies required in CHF discharge transition practice.

From a clinical perspective, the system provides a roadmap for competency development. CHF discharge planning is not a singular health education event but a continuous process encompassing assessment, education, communication, resource coordination, plan formulation, follow-up tracking, and quality improvement. Nurses may use this framework for comprehensive self-assessment of their role and capabilities in CHF discharge planning, thereby identifying targeted areas for improvement (35, 36). To mitigate the clinical burden of 52 items, high-weight indicators such as “II-1-3 Discharge risk assessment and stratification” and “II-4-1 Structured individualized discharge plan formulation” may be embedded into hospital information systems (HIS) at key workflow nodes, enabling automated data capture and reducing documentation workload. Lu et al. validated a 57-item scale with Cronbach's *α* = 0.98 among 1,075 nurses, demonstrating the feasibility of 50 + item instruments when supported by electronic administration (12).

From a management perspective, the system informs position evaluation and human resource allocation. Healthcare institutions may leverage this framework to assess nurses undertaking CHF discharge planning responsibilities, supporting position accreditation, tiered competency development, performance appraisal, and specialist nurse training (37, 38).

### Limitations

Several limitations warrant consideration. First, geographic and structural biases exist in expert representation. All 17 experts were based in China; no international experts were included, limiting the generalizability of findings. For instance, Koontalay et al. demonstrated in a Thai e-Delphi study that nurse-led CHF services in resource-constrained Southeast Asian settings prioritize community support and early decompensation response—a model substantially different from China's tertiary-hospital-centric, telephone-follow-up-dominant transitional care paradigm. Cross-cultural adaptation is therefore necessary before extrapolating this system to other national contexts. Additionally, the overrepresentation of nursing administrators (52.9%) may have attenuated the operational perspective of frontline nurses (39).

Second, content validity remains to be formally quantified (e.g., through content validity indices), and the system has not yet been pre-tested in an actual nurse population. Third-level Kendall's W values (0.158–0.292) indicated weak-to-moderate agreement, and both the Delphi and AHP methods inherently rely on subjective expert judgment.

Third, large-scale psychometric validation is still lacking. Unlike Lu et al., (who based their work on a validation study of 1,075 nurses in Ningxia with Cronbach's *α* = 0.98, this study has not yet conducted large-sample reliability and validity testing. The 52 third-level indicators have not been validated for clinical discrimination, inter-rater reliability, or test-retest reliability. Moreover, a 52-item instrument may impose considerable response burden in routine clinical settings. Although this study proposed a tiered usage strategy, it has not been empirically validated across different levels of healthcare institutions.

### Clinical feasibility of the 52-item system

The 52 third-level indicators constitute an “evaluation framework” rather than a “daily checklist.” A tiered approach is recommended: (1) for position accreditation, administer the 18 high-weight indicators (combined weight > 0.02); (2) for specialist nurse training, utilize the full set; (3) for information-enabled deployment, embed high-weight indicators such as II-1-3 (discharge risk assessment) and II-4-1 (structured discharge planning) into HIS nodes for automated data capture, thereby imposing no additional documentation burden on nurses. Lu et al.'s 57-item scale, validated in 1,075 nurses with Cronbach's *α* = 0.98, demonstrates the feasibility of 50 + item instruments under electronic administration. Future research will conduct factor analysis to derive an abbreviated version.

## Conclusion

Grounded in the iceberg model and employing a combination of literature review, the Delphi method, and the Analytic Hierarchy Process, this study constructed a competency evaluation index system for nurse-led CHF discharge planning. The final system comprises 5 first-level indicators, 16 s-level indicators, and 52 third-level indicators, demonstrating sound scientific rigor, reliability, and clinical utility. It provides a reference for nurse competency development, performance evaluation, and standardized management. Future research will focus on three priorities: (1) validating the scale's reliability and validity through multi-center studies and computing content validity indices (CVI); (2) developing standardized scoring manuals and interpretation guidelines for distinct use scenarios (training, assessment, self-evaluation); and (3) conducting cross-cultural adaptations across diverse healthcare systems to enhance generalizability.

## Data Availability

The original contributions presented in the study are included in the article/Supplementary Material, further inquiries can be directed to the corresponding author.
